# Primary synovial sarcoma in anal canal: Report of a very rare case

**DOI:** 10.1002/ccr3.9062

**Published:** 2024-06-11

**Authors:** Ramin Saadaat, Esmatullah Esmat, Jamshid Abdul‐Ghafar, Saif Ullah, Ahmed Nasir Hanifi, Abdul Latif Khairy, Ahmed Shekib Zahier, Ahmed Maseh Haidary, Sarah Noor

**Affiliations:** ^1^ Department of Pathology and Clinical Laboratory French Medical Institute for Mothers and Children (FMIC) Kabul Afghanistan; ^2^ Department of Internal Medicine French Medical Institute for Mothers and Children (FMIC) Kabul Afghanistan; ^3^ Central Public Health Laboratory, Ministry of Public Health Kabul Afghanistan; ^4^ Department of Oncology Amiri Medical Complex Kabul Afghanistan; ^5^ Department of Oncology Ali Abad Hospital Kabul Afghanistan

**Keywords:** anal canal, primary, sarcoma, Sinovial

## Abstract

Extensive studies are required to understand the behavior as well as prognosis of SS in the colorectal region. IHC staining is essential for the accurate diagnosis when a lesion is encountered at an unusual site.

## 1 INTRODUCTION

Synovial sarcoma (SS) represents a rare and unique subset of soft tissue malignant neoplasms that occurs in younger patients compared to other sarcomas. SS Mostly commonly affects adolescents and young adults, accounting for about 5%–10% of soft tissue sarcomas.^1^, ^2^ After rhabdomyosarcoma, SS is the second most common sarcoma and constitutes about 30% of childhood sarcomas.^1^

Most of the SS cases arises from joint capsule and articular tendons; however, it rarely involves the synovial structures and are thought to arise from pluripotential mesenchymal cells. Although not always apparently related to synovium, they have been called SS because of their histologic resemblance to synovium.^3^, ^4^

Synovial sarcoma most frequently develops in the lower extremities and less frequently affects the head, neck and trunk.^5^ In 2021, Zhang et al.^6^ reported a primary monophasic synovial sarcoma of rectum, and based on his literature review, at the time of his reporting only 70 cases of primary SS involving the gastrointestinal tract were reported. In this report, we present a case of a primary SS of anal canal, and to the best of our knowledge, no previous case of primary SS has been reported in anal canal.

## 2 CASE HISTORY/EXAMINATION

A 55‐year‐old man, farmer by profession, presented with 3 months complain of feeling a mass in the anal region, which was associated with painful defecation and occasional spotting of blood. On general physical examination, the patient was fine and healthy. Digital rectal examination demonstrated a mass in the anal canal, and the diagnosis of hemorrhoid was made, for which medical treatment including anti‐hemorrhoid local cream and analgesic were prescribed. Three months after the medical treatment, the patient showed no improvement in his symptoms. Thus, patient was referred for surgical evaluation with suspected neoplasm.

## 3 METHODS

**FIGURE 1:**
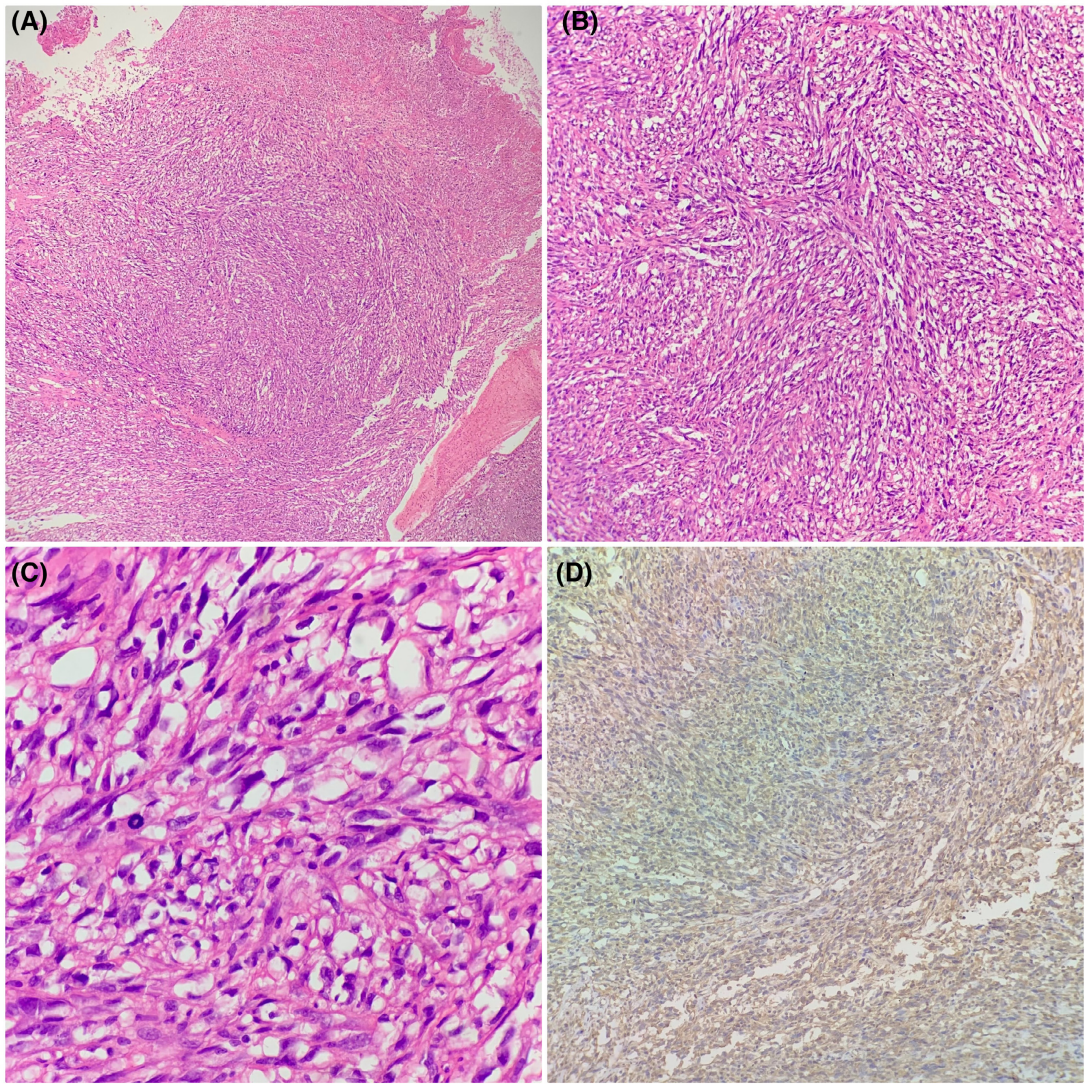
H&E staining of the tissue in 10\u00D7 magnification shows a spindle cell neoplasm arranged in sheets and nests (A). 20\u00D7 magnification, spindle cell neoplasm in hypercellular fascicular architecture with little intervening stroma (B). 40\u00D7 magnification, the neoplastic cells are large pleomorphic having abundant eosinophilic cytoplasm, hyperchromatic pleomorphic nuclei and mitotic figures (C). Ten magnification, Vimentin positive in neoplastic cells, shown in 10\u00D7 magnification (D).

**FIGURE 2:**
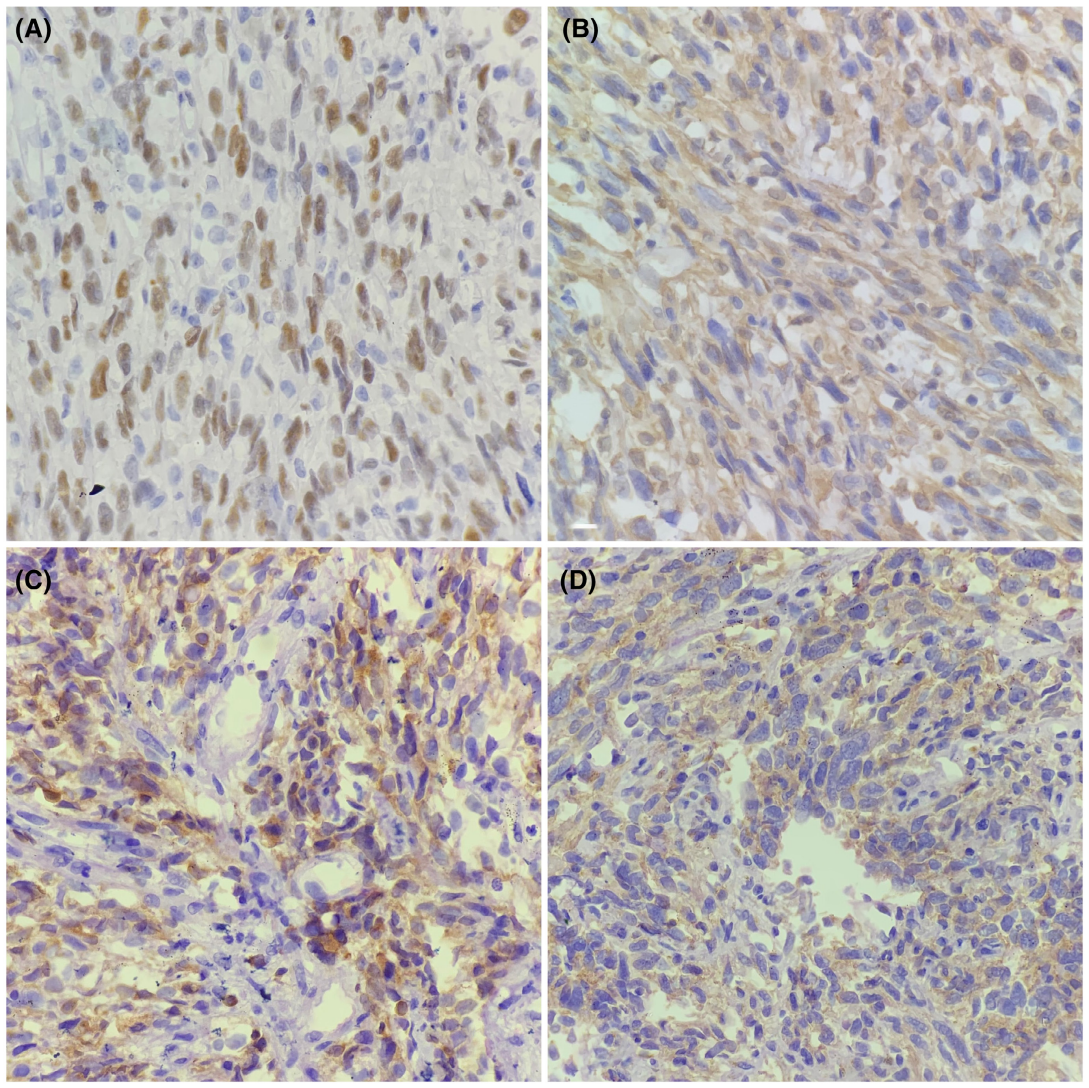
Immunohistochemical staining results in neoplastic cells; 40\u00D7 magnification, TLE1 which shows positivity in neoplastic cells (A, B). 40\u00D7 magnification, BCL2 which shows positivity in neoplastic cells (C). 40\u00D7 magnification, CD99 which shows positivity in neoplastic cells (D).

Accordingly, an incisional biopsy was performed and sent to the department of pathology of French Medical Institute for Mother and Children (FMIC) for histopathological evaluation. Grossly, the specimen was constituted by two small gray‐white tissue fragments. The tissue sections prepared from the paraffin‐fixed blocks were stained with Hematoxylin and Eosin (H&E). Microscopic examination of the HE‐stained sections demonstrated a mucosal ulceration and a spindle cell neoplasm arranged in haphazard pattern. The neoplastic cells were atypical, having enlarged hyperchromatic nuclei and prominent nucleoli. Many mitotic figures were also noted, (Figure 1A–C). Immunohistochemical (IHC) stains were performed in which the neoplasm showed positive staining for Vimentin (Figure 1D), TLE1, CD99, and BCL2 (Figure 2A–D), while it was negative for Pan Cytokeratin, CD34, S100 protein, HMB45, SMA, Desmin, Myogenin, STAT6, and DC117.

Based on its H&E morphology and IHC stains, the diagnosis of synovial sarcoma was concluded. Since the past medical and surgical history of the patient was unremarkable and the physical examination and computed tomography (CT) scan showed no mass lesion in soft tissue, the possibility of metastatic SS from soft tissue origin was ruled out.

## 4 CONCLUSION AND RESULTS

Eight months after the diagnosis, we enquired about the patient's status and course of treatment and were informed that he had received multiple courses of chemotherapy and had not had surgery based on oncologists decision. Unfortunately, due to very limited oncology centers in our country, the patient was advised to go to one of our neighboring country for chemotherapy. Thus, so we cannot collect exact information about type of chemotherapy and number of cycles. Furthermore, a recent CT scan revealed lung and liver metastases.

## 5 DISCUSSION

Predominant malignant neoplasms of colon, rectum, and anus are epithelial malignancies, including adenocarcinoma and squamous cell carcinoma, and rarely can be affected by sarcomas, which constitutes less than 0.1% of all malignant neoplasms in the lower gastrointestinal tract.^7^ Amongst sarcomas, the leiomyosarcoma is the most common type of colon sarcoma.^8^

Synovial sarcoma is a rare and high‐grade soft tissue malignant neoplasm that makes 6% of all sarcomas. It has three histologic variants: 1. Monophasic 2. Biphasic and poorly differentiated.^5^ Monophasic synovial sarcoma is composed of only spindle‐shaped neoplastic cells, while biphasic SS contain mixtures of spindle‐shaped neoplastic cells and epithelial neoplastic cells.^9^ SS can occur at any age but commonly occur in third decades of life. SS most commonly involve lower extremities followed by upper extremities, head, neck, and trunk.^10^ SS with well‐differentiated morphology in patients under 25 years of age and tumor size less than 5 cm are considered as low‐risk, while patients over 25 years of age with tumors that are poorly differentiated and more than 5 cm in size are categorized as high‐risk patients.^5^ Primary SS of the gastrointestinal tract is very rare, and only 70 cases have so far been reported in the literature.^8^ To the best of our knowledge with extensive literature review, no case of primary SS originating in the anal region has been reported in literature, and our current case was the first case of primary SS arising in anal canal of an elder male. Correct diagnosis of SS in such uncommon location is very important and should be differentiated from carcinomas, common sarcomas of the region including leiomyosarcoma and gastrointestinal stromal tumor (GIST). Our case was clinically diagnosed as hemorrhoid that delayed the accurate diagnosis. The biopsy tissue microscopic examination showed malignant spindle cell neoplasm that could indicate the differential diagnoses of GIST, leiomyosarcoma, other types of sarcoma, and poorly differentiated carcinoma with sarcomatoid changes. GIST had to be positive for CD117 IHC and negative for TLE1, leiomyosracoma and rhabdomyosarcoma must express desmin, AMA, and Myogenin IHC stains and would be negative for TLE1. Poorly differentiated carcinoma must be positive for pan cytokeratin and negative for Vimentin.

It is important to differentiate colorectal SS from its mimicking neoplasms. Leiomyosarcoma is very rare in colon, but it is most common sarcoma of the colon and constitutes about 90% of colorectal sarcomas. It should be remembered that GIST is not included in primary sarcomas of colon.^11^ GIST is most common in stomach and small intestine but can rarely involve the colorectal region. Leiomyoma and leiomyosarcoma show positivity for ASMA and desmin. GIST are positive for CD117 (c‐Kit) and CD34.^12^ In our case, Desmin, ASMA, and CD117 were negative. The Transducin‐Like Enhancer (TLE) of split genes encode human transcriptional corepressors that are involved in embryogenesis and hematopoiesis. Gene expression profiling studies have consistently shown the TLE family of genes, TLE1 in particular, is demonstrated to be overexpressed in the nuclei of synovial sarcoma cells.

Most synovial sarcomas are characterized by a specific chromosomal t(X;18)(p11;q11) translocation that leads to fusion of the SS18 gene on chromosome 18 with an SSX partner on chromosome X, frequently SSX1 and SSX2, and rarely SSX4. Therefore, the identification of t(X;18)(SS18;SSX) is considered the gold standard for the diagnosis of synovial sarcomas, and t(X;18)(SS18;SSX) can be detected by fluorescence in situ hybridization (FISH), reverse transcriptase‐polymerase chain reaction (RT‐PCR), or cytogenetics. However, molecular studies have not been widely used in all laboratories.^13^, ^14^ Gene expression profiling studies have consistently shown expression of TLE family of genes, TLE1 in particular, in the nuclei of synovial sarcoma cells. In our case, the neoplastic cells showed nuclear positivity for TLE1; therefore, the high possibility of SS was made, for which further molecular studies were suggested for confirmation.

Epithelial components of SS can be positive for pan cytokeratin, epithelial membrane antigen (EMA), CAM 5.2 immunostains. At least one of the mentioned IHC stain will be positive in a case of SS. However, these markers will show less strong reactivity in spindle cell components of SS. In few cases, if there is positive staining for epithelial markers, it should be very patchy in contrast to carcinoma, which shows strong and diffuse reactivity with epithelial markers.^15^ Bcl‐2 is positive in synovial sarcoma and initially was thought to be a specific marker for this tumor.^16^ Subsequent studies, however, have shown that a significant proportion of other tumors in the differential diagnosis of synovial sarcoma (solitary fibrous tumors, malignant peripheral nerve sheath tumors, and mesothelioma) are Bcl‐2 positive, minimizing the utility of this antibody.^15^ CD99 is positive in about 60% of synovial sarcomas, which can cause confusion with Ewing's/primitive neuroectodermal tumor, especially in small samples or poorly differentiated synovial sarcomas. Proper histopathological evaluation with IHC staining is essential for the accurate diagnosis when a lesion is encountered at an unusual site. Extensive studies are recommended to elaborate further information regarding clinical features and prognosis of SS in the colorectal region. To the best of our knowledge, this was the first case of SS in the anal canal.

**Table:** 

Patient perspective: The patient stated that it would be useful to publish and share this case with other healthcare workers, to enable better understanding and correct diagnosis of such rare cases that would cause a specific treatment and follow up.

## AUTHOR CONTRIBUTIONS

**Ramin Saadaat:** Conceptualization; investigation; validation; visualization; writing – original draft; writing – review and editing. **Esmatullah Esmat:** Investigation; validation. **Jamshid Abdul‐Ghafar:** Investigation; supervision. **Saif Ullah:** Investigation; methodology. **Ahmed Nasir Hanifi:** Resources; supervision. **Abdul Latif Khairy:** Resources. **Ahmed Shekib Zahier:** Validation; visualization; writing – review and editing. **Ahmed Maseh Haidary:** Supervision; writing – original draft; writing – review and editing. **Sarah Noor:** Resources; software; supervision; visualization.

## FUNDING INFORMATION

None.

## CONFLICT OF INTEREST STATEMENT

The authors declare to have no conflict of interest.

## ETHICAL APPROVAL

Ethical approval was obtained from the hospital's ethical review committee.

## CONSENT

Written informed consent was obtained from the patient to publish this report in accordance with the journal's patient consent policy.

## Data Availability

All generated data is included in this article.
